# The ANA-reflex test as a model for improving clinical appropriateness in autoimmune diagnostics

**DOI:** 10.1007/s13317-016-0080-3

**Published:** 2016-07-16

**Authors:** Elio Tonutti, Nicola Bizzaro, Gabriella Morozzi, Antonella Radice, Luigi Cinquanta, Danilo Villalta, Renato Tozzoli, Marilina Tampoia, Brunetta Porcelli, Martina Fabris, Ignazio Brusca, Maria Grazia Alessio, Giuseppina Barberio, Maria Concetta Sorrentino, Antonio Antico, Danila Bassetti, Desré Ethel Fontana, Tiziana Imbastaro, Daniela Visentini, Giampaola Pesce, Marcello Bagnasco, On behalf of the Study Group on Autoimmune Diseases of the Italian Society of Clinical Pathology and Laboratory Medicine

**Affiliations:** 1 Laboratory of Immunopathology and Allergology, University Hospital Udine, P.le S. Maria della Misericordia 15, 33100 Udine, Italy; 2 Laboratory of Clinical Pathology, San Antonio Hospital, Tolmezzo, Italy; 3 Rheumatology Section, Department of Medical, Surgical and Neuro Sciences, University of Siena, Siena, Italy; 4 Microbiology and Virology, San Carlo Borromeo Hospital, Milan, Italy; 5 Laboratory of Clinical Pathology, University Hospital San Giovanni di Dio and Ruggi D’Aragona, Salerno, Italy; 6 Clinical Allergology and Immunology, S. Maria degli Angeli Hospital, Pordenone, Italy; 7 Laboratory of Clinical Pathology, S. Maria degli Angeli Hospital, Pordenone, Italy; 8 Clinical Pathology I, University Hospital Policlinico Consorziale, Bari, Italy; 9 Department of Medical Biotechnology, University of Siena, Siena, Italy; 10 Analysis Laboratory, Buccheri La Ferla Hospital, Palermo, Italy; 11 Clinical Chemistry Analysis Laboratory, Papa Giovanni Hospital XXIII, Bergamo, Italy; 12 Laboratory Medicine, Department of Clinical Pathology, Treviso Hospital, Treviso, Italy; 13 Clinical Chemistry and Microbiology Analysis, ISMETT, Palermo, Italy; 14 Analysis Laboratory, Santorso, Italy; 15 Microbiology and Virology, S. Chiara Hospital, Trento, Italy; 16 Postgraduate School of Clinical Pathology and Biochemistry, University of Padua, Padua, Italy; 17 Laboratory of Autoimmune Diseases, P.O. Spirito Santo, Pescara, Italy; 18 DIMI, University of Genova, Genoa, Italy

**Keywords:** Antinuclear antibodies, ANA, Reflex test, Immunofluorescence, Pattern, Intracellular specific antigens

## Abstract

Reflex tests are widely used in clinical laboratories, for example, to diagnose thyroid disorders or in the follow-up of prostate cancer. Reflex tests for antinuclear antibodies (ANA) have recently gained attention as a way to improve appropriateness in the immunological diagnosis of autoimmune rheumatic diseases and avoid waste of resources. However, the ANA-reflex test is not as simple as other consolidated reflex tests (the TSH-reflex tests or the PSA-reflex tests) because of the intrinsic complexity of the ANA test performed by the indirect immunofluorescence method on cellular substrates. The wide heterogeneity of the ANA patterns, which need correct interpretation, and the subsequent choice of the most appropriate confirmatory test (ANA subserology), which depend on the pattern feature and on clinical information, hinder any informatics automation, and require the pathologist’s intervention. In this review, the Study Group on Autoimmune Diseases of the Italian Society of Clinical Pathology and Laboratory Medicine provides some indications on the configuration of the ANA-reflex test, using two different approaches depending on whether clinical information is available or not. We further give some suggestions on how to report results of the ANA-reflex test.

## Introduction

The term reflex test indicates a “cascade” diagnostic approach where a positive initial (first level) test automatically triggers further (second level) tests based on predefined rules applied to information systems. Cascade algorithms have been used for some time in autoimmune diagnostics, in particular for the detection of anti-nuclear-cytoplasmic antibodies (ANA) [1–3], but in spite of its obvious contribution in terms of diagnostic appropriateness, the ANA-reflex test is not yet widely implemented [4, 5]. As we shall see shortly, this is related to the complexity of the diagnostic algorithm of the ANA-reflex test which does not rely on informatics automatism, but rather on the intervention of a pathologist based on clinical information and preceding results [6], and should, in fact, be more appropriately defined “ANA-reflective” testing [7]. Be that as it may, for simplicity, custom, and convenience, in this text, we will refer to “ANA-reflex” testing.

The ANA-reflex test differs from other current laboratory reflex tests both conceptually and organizationally. For example, the thyroid-stimulating hormone (TSH)-reflex test relies on the sequential execution of specific tests, inserted into a well-defined algorithm based on the TSH test result, without the need for decisional intervention by the operators. ANA-reflex testing is certainly more complex than TSH-reflex or other reflex testing for several reasons. First and foremost, ANA testing has a very low predictive value. Second, and by no means less importantly, ANA is a first-level test not for the diagnosis of a sole condition, but for several systemic autoimmune rheumatic diseases (systemic lupus erythematosus, mixed connective tissue disease, undifferentiated connective tissue disease, Sjögren’s syndrome, and scleroderma), as well as autoimmune hepatic disorders. Third, ANA is detected by indirect immunofluorescent antibody assay (IIF), a subjective interpretative assay, with all the associated variables applicable to this type of method. Furthermore, ANA testing by IIF is complicated by the number of positive patterns attainable (>50), which necessitates interpretation by a pathologist and requires appropriate confirmation tests. Another peculiar characteristic is that ANA testing at the dilution of 1:40 in IIF can be positive in up to 20–30 % of healthy subjects, and finally, certain positive ANA patterns, like *dense fine speckled* or DFS70 if monospecific, are not associated with systemic autoimmune disorders even at high titres [8–10].

For these reasons, the introduction of an ANA-reflex test is an intriguing challenge both in terms of approach and algorithm construction. Whichever these difficulties should not impede the application of ANA-reflex testing considering its undeniable advantages. ANA-reflex testing could, indeed, be useful to the general practitioner or to the non-rheumatology specialist who entrusts the seroimmunological investigation of a patient with a potential systemic autoimmune rheumatic disorder to the laboratory. The objective is to simplify the patient work-up: a single visit to the doctor’s surgery, a single visit to the laboratory, and thus a more rapid clinical diagnosis.

The economic implications of ANA-reflex testing would be very relevant if its application lead to a reduction of second-level tests, e.g., antibodies to intracellular-specific antigens (so-called ENA) and anti-dsDNA. At present, laboratories in some jurisdictions are in fact “obliged” to execute these second-level tests on demand, irrespective of the ANA test result, which leads to increased spending in the absence of any clinical and diagnostic justification [3].

This document proposes one ANA-reflex algorithm to confirm a diagnosis of an ANA-associated rheumatic disease (AARD) based exclusively on the laboratory result for laboratories without access to clinical information, and another based on both laboratory results and clinical information. These two algorithms then merge into a common pathway.

## ANA-reflex test procedure with titres ≥1:160 and typical patterns

Table 1 indicates which reflex tests should be executed based on the pattern type observed on the HEp-2 cells. The evaluation of the ANA test pattern is fundamental to the execution of the second-level tests. The specific autoantibodies responsible for typical ANA patterns are clearly described in the literature [11–15] and for certain fluorescent patterns, such as homogeneous, speckled, fine grainy (Scl70-like), nucleolar, centromeric or speckled cytoplasmic, the identification of precise autoantibody markers is considered essential, while for others it is not deemed to be necessary. The second-level testing for antibodies to intracellular specific antigens involves a screening test for antibodies directed against the classical antigens (Ro60 and Ro52, La, Sm, RNP, Jo1, CENP-B, Scl70, and dsDNA). This selection is based on the fact that the antibodies directed against these antigens are more frequently associated with autoimmune rheumatic diseases, and the tests are readily available commercially.

**Table 1:** ANA-reflex test procedure with titres ≥1:160 and typical patterns

ANA-IIF pattern on HEp-2 cells	Reflex test(s)
Nuclear homogeneous ≥1:160	Antibodies to intracellular specific antigens (ENA) and to dsDNA/nucleosomes
Nuclear speckled ≥1:160	Anti-dsDNA and antibodies to intracellular specific antigens (ENA), possibly including anti-RNA polymerase III
Nuclear Scl70-like ≥1:160	Antibodies to intracellular specific antigens (ENA) (possibly including anti-PM/Scl)
Cytoplasmic speckled ≥1:160	Antibodies to intracellular specific antigens (ENA), including anti-tRNA synthetases and anti-P ribosomal
Pleomorphic PCNA-like (any titre)	Anti-PCNA
Centromere	No confirmation necessary if high titres. Execute specific test for anti-CENP B only in dubious cases (low titre or centromeric pattern not clearly recognizable)

ENA includes SS-A/Ro52 and Ro60, SS-B/La, Sm, RNP, Jo-1, and Scl70

The use of the HEp-2 cell line for the execution of the ANA test allows for the identification of numerous other patterns defined as rare, cytoplasmic or in cellular replication phase that may, in selected cases, provide the clinician with useful information. In most cases, these patterns do not require further testing inasmuch as the antigenic target is neither known nor confirmable with specific tests. Accordingly, confirmation tests are not indicated for the following patterns: a few nuclear dots, low titre nucleolar (<1:160), spindle fibers, NuMa-like, intercellular bridge, CENP-F-like, cytoplasmic GW bodies, polar/Golgi-like, and cytoplasmic filamentous/microtubules.

## ANA-reflex test procedure with dense fine speckled-DFS70 pattern

The DFS70 pattern deserves particular consideration, since recent evidence highlighted it as one of the most frequent findings in ANA-IIF testing. From a morphological perspective, the DFS70 pattern is well characterized: HEp-2 cell presents fairly course granular fluorescence of the nuclei sparing the nucleoli, while the chromatinic region of mitotic cells is intensely fluorescent, maintaining the typical granularity. This pattern should urge the pathologist to perform a confirmation test to identify anti-DFS70 specificity [16]. If isolated anti-DFS70 is confirmed in the absence of signs and symptoms suggestive of AARD, the pathologist should indicate in his/her report that the evidenced ANA pattern, even at very high titres, is generally not indicative of AARD.

In the event that the execution of a specific anti-DFS70 test is not possible, it is recommended that a descriptive comment of the pattern is inserted on the report along with any possible diagnostic correlations.

It goes without saying that whenever signs and symptoms of autoimmune rheumatic disease are present, anti-dsDNA and anti-intracellular specific antigen antibodies should be tested, even in the presence of an anti-DFS70 pattern. The anti-DFS70 pattern at high titre might in fact “mask” ANA positivity with a different pattern [16].

Subsequently, we suggest an approach to the further steps necessary to diagnose ANA-reflex test in subjects who were identified as symptomatic by the requesting clinician. This should not be considered if the laboratory does not have access to clinical information.

## Indications for ANA-reflex testing supported by clinical information

In our opinion, it would be useful if the ANA-reflex test request was accompanied by clinical information, since some signs and symptoms could independently justify the execution of the second-level tests [17]. The exact nature of the signs and symptoms to associate to the ANA-reflex test request should be decided in conjunction with the clinical specialists (rheumatologists). Out of the classification criteria for the respective AARDs, we have identified the following clinical findings that could warrant the second-level tests even in the case of low-titre ANA positivity or ANA negativity: Raynaud’s phenomenon, photosensitivity or malar rash, persistent oral or ocular dryness, leucopenia or lymphopenia, significant increase in the creatine phosphokinase (CPK) enzyme, persistent arthritis, thrombotic events, or recurrent miscarriages. Some of the aforementioned clinical findings are subjective, but nonetheless relevant in the suspicion of AARDs.

In the presence of these conditions, the pathologist should react, as shown in Table 2.

**Table 2:** ANA-reflex test procedure in relation to clinical manifestations

Clinical manifestation	Reflex test(s)
Persistent oral or ocular dryness	Anti-intracellular specific antigens (anti-ENA)
Raynaud’s phenomenon and/or photosensitivity (or malar rash) and/or leucopenia and/or arthritis	Antibodies to dsDNA and to intracellular specific antigens (anti-ENA)
Raynaud’s phenomenon and ANA positivity with nucleolar pattern at elevated titres (≥1:320)	Anti-PM/Scl, anti-fibrillarin, anti-RNA polymerase III and Th/To
Significantly increased CPK	Antibodies to intracellular specific antigens (anti-ENA) and myositis-associated antibodies
ANA positivity (even at a titre 1:80) and persistent arthritis	Anti-citrullinated peptide antibodies and rheumatoid factor
Positive ANA and/or SLE-associated specific antibodies (dsDNA, Sm, RNP, Ro52, and 60Kd), with a clinical history of thrombotic events and/or polyabortion	Anti-phospholipid antibodies (anti-cardiolipin, anti-beta2 glycoprotein I, lupus anticoagulant)

The laboratory is able to identify a much larger number of autoantibodies that can be found in various autoimmune pathologies with varying frequency. The identification methods, in general, are immunoblot or microarray that in some countries currently present such elevated costs as to be used only in selected cases. We believe therefore that such diagnostic investigations are justified only in a specialized setting. Consequently, it is not appropriate to integrate these investigations into the ANA-reflex algorithm.

An additional consideration regards the capacity of a positive ANA test to predict uveitis in juvenile idiopathic arthritis (JIA) or to evidence autoantibodies that correlate with autoimmune hepatitis. Widespread use of the ANA-reflex test for diagnosing such pathologies, however, is not advisable considering that only some of the markers for autoimmune hepatitis can be identified by ANA-IIF on HEp-2 cells. Nevertheless, in the presence of a pattern suggestive of an autoimmune hepatitis-associated marker, confirmation tests are indicated. Table 3 proposes a correct diagnostic procedure in the case of positivity for this group of autoantibodies.

**Table 3:** ANA-reflex test procedure with patterns related to markers found in autoimmune liver diseases

ANA-IIF pattern on HEp-2 cells	Reflex test(s)
Cytoplasmic reticular/AMA ≥1:160	Anti-mitochondrial M2 or E3 or MIT3
Multiple nuclear dots ≥1:160	Anti-Sp100
Nuclear envelope	Anti-gp210
Cytoplasmic linear-actin ≥1:160	IIF on kidney, stomach, and liver, to confirm anti-actin antibodies

## The ANA-reflex test report

An interpretative comment on the ANA-reflex report is important, and should include an explanation of the results obtained as well as the possible diagnostic route undertaken [18]. For example, in the presence of an unexpected marker for autoimmune hepatitis, it should be indicated that the finding of such autoantibodies “could be associated with autoimmune hepatitis.” In the presence of anti-DFS70 antibodies (possibly confirmed with specific tests), it should be indicated that said marker “does not generally correlate with ANA-associated autoimmune pathology.” When the second-level tests are executed in the context of ANA negativity, the reason for following that particular diagnostic procedure should be explained.

## Administrative aspects of the ANA-reflex test

The proposal of the SIPMeL study group wants merely to be a referral model in terms of type and modality of the second-level test execution. From our group’s proposal, it is evident that any patient with ANA-reflex could have his own more or less complex course, in some cases articulated with more second-level tests. This, peculiarity, should not translate to difficulty of the bureaucratic or administrative type: in fact, it is not conceivable that the ANA-reflex test requires a tariff calculation for each request. In Italy, the cost of reflex tests is predetermined on the basis of an approximate calculation of the number and type of further tests that could be executed. This way, at the moment of administrative procedure, the patient with the ANA-reflex test request is charged a flat rate, which will cover all eventual further tests. That allows the elimination of complex administrative procedures associated with additional requests or payments. This model may, of course, be applied differently in other countries, according to local laws or regulations [19].

One final consideration, befitting the context in which laboratories operate, is that if, on the one hand, the adoption of the ANA-reflex request modality is aimed at improving the handling of resources, it is also and above all a cultural application able to provide rapid complete diagnostic information with important repercussions on subsequent clinical decision.

## References

[1] Homburger HA (1995). Cascade testing for autoantibodies in connective tissue diseases. Mayo Clin Proc.

[2] Bizzaro N, Wiik A (2004). Appropriateness in anti-nuclear antibody testing: from clinical request to strategic laboratory practice. Clin Exp Rheumatol.

[3] Bizzaro N, Morozzi G (2009). A proposed model for effective collaboration between rheumatologists and clinical pathologists for the diagnosis of autoimmune rheumatic diseases. Rheumatol Int.

[4] Agmon-Levin N, Damoiseaux J, Kallenberg C (2014). International recommendations for the assessment of autoantibodies to cellular antigens referred to as anti-nuclear antibodies. Ann Rheum Dis.

[5] Wiik AS, Bizzaro N (2012). Missing links in high quality diagnostics of inflammatory systemic rheumatic diseases. Autoimmun Highlights.

[6] Tampoia M, Brescia V, Fontana A (2007). Application of a combined protocol for rational request and utilization of antibody assays improves clinical diagnostic efficacy in autoimmune rheumatic disease. Arch Pathol Lab Med.

[7] Shrivastava R, BartlettWA Kennedy IM (2010). Reflex and reflective testing: efficiency and effectiveness of adding on laboratory tests. Ann Clin Biochem.

[8] Mariz HA, Sato EI, Barbosa SH (2011). Pattern on the antinuclear antibody-HEp-2 test is a critical parameter for discriminating antinuclear antibody-positive healthy individuals and patients with autoimmune rheumatic diseases. Arthritis Rheum.

[9] Fitch-Rogalsky C, Steber W, Mahler M (2014). Clinical and serological features of patients referred through a rheumatology triage system because of positive antinuclear antibodies. PLoS One.

[10] Fabris M, Zago S, Tosolini R (2014). Anti-DFS70 antibodies: a useful biomarker in a pediatric case with suspected autoimmune disease. Pediatrics.

[11] Stinton LM, Fritzler MJ (2007). A clinical approach to autoantibody testing in systemic autoimmune rheumatic disorders. Autoimmun Rev.

[12] Wilk A, Hoier-Madsen M, Fordlid J (2010). Antinuclear antibodies: a contemporary nomenclature using HEp-2 cells. J Autoimmun.

[13] Vermeersch P, Bossuyt X (2013). Prevalence and clinical significance of rare antinuclear antibody patterns. Autoimmun Rev.

[14] Chan EKL, Damoiseaux J, Carballo OG (2015). Report of the first international consensus on standardized nomenclature of antinuclear antibody HEp-2 cell patterns 2014–2015. Front Immunol.

[15] Damoiseaux J, von Muhlen CA, Garcia-De la Torre I et al (2016) International consensus on ANA pattern (ICAP): the bumpy road towards a consensus on reporting ANA results. Autoimmun Highlights 7:1. doi:10.1007/s13317-016-0075-0 10.1007/s13317-016-0075-0 PMC4733811 26831867

[16] Bizzaro N, Tonutti E, Tampoia M (2015). Specific chemoluminescence and immunoasdorption tests for anti-DFS70 antibodies avoid false positive results by indirect immunofluorescence. Clin Chim Acta.

[17] Wiik AS (2004). Cutting edge diagnostics in rheumatology: on the role of patients, clinicians, and laboratory scientists in optimizing the use of autoimmune serology. Arthritis Care Res.

[18] Tonutti E, Visentini D, Bizzaro N (2007). Interpretative comments on autoantibody tests. Autoimmun Rev.

[19] Fritzler MJ (2016). Choosing wisely: review and commentary on anti-nuclear antibody (ANA) testing. Autoimmun Rev.

